# A bespoke, real-time surveillance system for malaria elimination in Cambodia: architecture, applications and impact

**DOI:** 10.1186/s12936-025-05772-1

**Published:** 2026-01-12

**Authors:** Pengby Ngor, Huy Rekol, Siv Sovannaroth, Thyda Eng, Rith Ry, Kimleng Sok, Rattana Yoem, Vunsokserey Ou, Vanna Hem, Pumeoung Chang, Rady Try, Pascal Ringwald, Lisa J. White, Richard J. Maude

**Affiliations:** 1 National Center for Parasitology, Entomology and Malaria Control, No 372, Preah Monivong, Corner Street 322, Phnom Penh, Cambodia; 2 The Open University, Milton Keynes, UK; 3 Mahidol Oxford Tropical Medicine Research Unit, Faculty of Tropical Medicine, Mahidol University, Bangkok, Thailand; 4 WHO Mekong Malaria Elimination Programme, Phnom Penh, Cambodia; 5 Model Health Ltd, Brixham, UK; 6 Centre for Tropical Medicine and Global Health, Nuffield Department of Medicine, University of Oxford, Oxford, UK

**Keywords:** Malaria, Surveillance, Cambodia, Digital health, Case management, Real-time data, Mobile health, Elimination, Village malaria workers, Malaria information system

## Abstract

Cambodia has developed and deployed a comprehensive, real-time, case-based Malaria Information System (MIS) to support national malaria elimination goals. This locally built, user-centred digital platform integrates surveillance, diagnostics, treatment, logistics, entomology and operational monitoring into a single system accessible at all levels of the health system. Mobile and web-based applications enable village malaria workers and health facility staff to report cases and interventions in real time, even in offline settings. The system promotes decentralized decision-making through intuitive dashboards and customizable analytics, enhancing local ownership, accountability and responsiveness. It supports key strategies such as the 1–3-7 surveillance model, integrated drug efficacy surveillance and incidence-based stratification. Operational functionality and system sustainability is supported by MIS modules for device management, stock management, geolocation, training and quality assurance. Real-time analytics drive timely interventions and adaptive planning, while interoperability with regional and global databases facilitates cross-border coordination and external reporting. Challenges remain, such as limited technical support capacity and a need for predictive tools. However, the Cambodia MIS demonstrates that locally developed digital health systems can transform disease surveillance and accelerate elimination efforts when effectively integrated with community-based networks and supported by strong governance. This model provides valuable evidence for other countries aiming to transition from malaria control to elimination, while complying with elimination certification requirements, and preparing for the prevention of re-establishment of transmission once malaria elimination has been achieved.

## Introduction

Historically, Cambodia has experienced a substantial malaria disease burden and been a focus for the emergence of antimalarial drug resistance [1]. The country is highly receptive to malaria transmission, particularly in the forested border regions with Lao People’s Democratic Republic (PDR), Thailand and Viet Nam, and harbours a range of Anopheles mosquito vectors for both *Plasmodium falciparum* and *Plasmodium vivax* [2].

*P. falciparum* partial resistance to artemisinin was first reported in western Cambodia in 2006 [3], and spread rapidly across the Greater Mekong Sub-region (GMS) [4]. Clinical outcomes were impacted by the subsequent emergence of multi-drug-resistant parasites [5, 6]. The threat of these strains spreading globally precipitated an emergency response from the World Health Organization (WHO) [7], and the drive for malaria elimination in the GMS by 2030 [8].

**Fig. 1:**
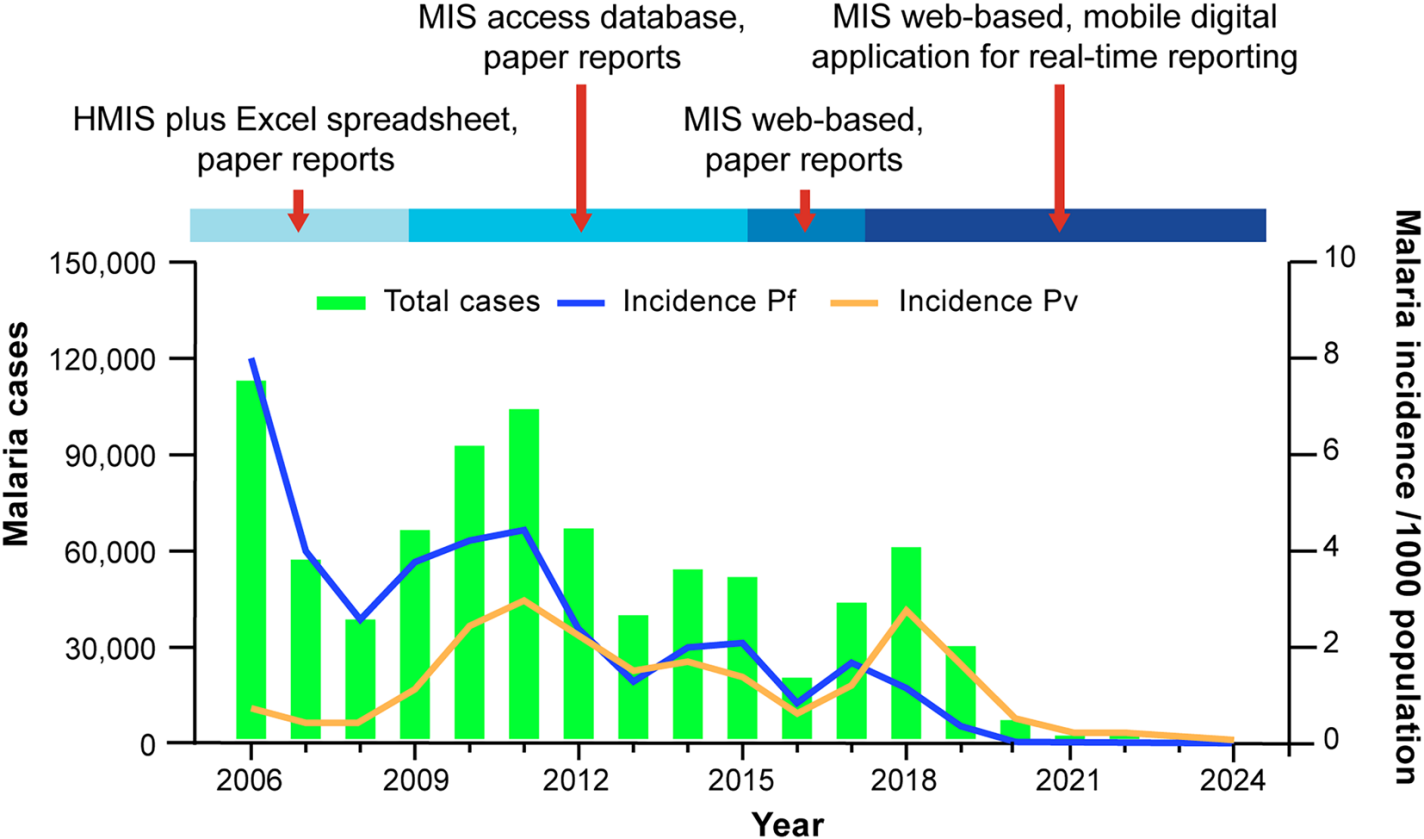
Evolution of malaria surveillance in Cambodia to support elimination. Transitioning to the elimination phase required surveillance strengthening, evolving from a paper-based reporting system to a fully digital system with real-time mobile reporting. Source: CNM/MIS. HMIS, health management information system; Pf, *P. falciparum* malaria; Pv, *P. vivax* malaria; MIS, malaria information system. Malaria incidence is shown as per 1000 population

In 2011, Cambodia launched its strategic plan for malaria elimination by 2025 [9]. Supported by the malaria elimination action frameworks, 2016–2020 and 2021–2025 [10], the country has made significant progress towards this goal, with annual cases decreasing by 99.7% from 106,228 in 2010 to just 355 in 2024 [11]. Along with rapid case management and vector control, a key pillar of this process was the establishment of comprehensive, effective and timely case-based surveillance (Fig. 1).

Before 2009, malaria case data from health facilities were available through the National Health Management Information System (HMIS), aggregated at the operational district level. In 2004, the village malaria worker (VMW) network was launched to provide malaria diagnosis and artemisinin-based combination therapy in villages located > 5 km from the nearest health centre [12]. The National Center for Parasitology, Entomology and Malaria Control (CNM) launched a parallel surveillance system, with VMWs recording individual case data on paper forms and manual aggregation by CNM using Microsoft Excel (Microsoft Corporation, Redmond, USA). Both the HMIS and CNM systems were limited by incomplete and delayed data, and neither supported village-level risk stratification. Notably, the 1–3–7 strategy to support malaria elimination – requiring case notification within 1 day, investigation within 3 days, and response within 7 days – could not be effectively operationalized with these systems [13].

In 2009, the National Malaria Control Programme introduced the Malaria Information System (MIS) to support operational district staff in overseeing malaria control efforts, including insecticide-treated bed net distribution and data collection from VMWs. The MIS operated independently from the HMIS as a standalone Microsoft Access database, tailored to the country’s limited internet connectivity at the time [14, 15]. Data were collected on paper forms and aggregated at the operational district level with automatically generated monthly updates including incidence maps at the village level, allowing malaria risk re-stratification. By 2013, additional functionalities were available, including case-based reporting and provincial and national data outputs. In 2015, the MIS was upgraded to a web-based platform [14, 15], with monthly paper reports submitted by health facilities and manual data entry at the operational district level. Challenges persisted, including low data completeness and system complexity, requiring specialised training at the provincial and district levels. The system did not provide real-time data from VMWs and health facilities, and its visual outputs were inadequate for elimination planning. Furthermore, the system was unable to generate key indicator reports, further limiting effectiveness.

Transitioning from malaria control to elimination requires a strong surveillance system with comprehensive, real-time, case-based data reporting and intervention mapping to quickly detect infections and disrupt transmission [16, 17]. Existing systems, such as DHIS2, did not address these needs, providing only aggregated data which did not meet the criteria for malaria elimination − a malaria module was not released until 2021, and a logistic support module was not available before 2020 [18]. As a web-based platform, DHIS2 was not feasible as the basis of an integrated community-based malaria elimination surveillance system because of inadequate digital infrastructure. Although a mobile reporting app was available from 2014, offline capabilities were not introduced until 2018 and the app was not optimized for low e-literacy or for community-based interventions. However, mobile reporting had been piloted in several provinces in Thailand in 2009, with the aim of targeting border malaria [19]. Such initiatives provided valuable evidence of the potential for mobile reporting to increase data flow from rural and remote locations as well as useful lessons learned [20].

In Cambodia, achieving elimination capable surveillance goals required innovative, context-appropriate solutions, with a focus on feasibility, affordability and sustainability. In response, the CNM launched an enhanced MIS in 2017, including mobile applications optimized for VMWs and health centre staff enabling them to report individual, geolocated malaria cases directly from the field in real time (https://mis.cnm.gov.kh). The bespoke system was developed locally by CNM, with financial support from the Global Fund. In this paper, we describe the development and functionality of the enhanced MIS and highlight its impact on malaria surveillance and operational outcomes.

## Bespoke real-time surveillance system for malaria elimination

### Architecture and development

#### Development team

The MIS team comprises seven key personnel. One member serves as the chief technical officer overseeing the broader malaria control and elimination programme (SS). The MIS team is led by one individual (PN), who manages all MIS operations, including data oversight, system feature specifications and system implementation. Support staff include two software developers, responsible for the design, development and maintenance of the web-based MIS platform (SK, YR); two data management officers who address user feedback, verify data completeness and timeliness and ensure data accuracy (OV, HV); and a sub-team of three individuals maintain both the web-based and Android-based mobile applications (ET, RR, CP). Having the entire system developed and maintained by a local Cambodian team minimises costs, facilitates rapid updates and helps ensure long term sustainability.

#### System architecture

**Fig. 2:**
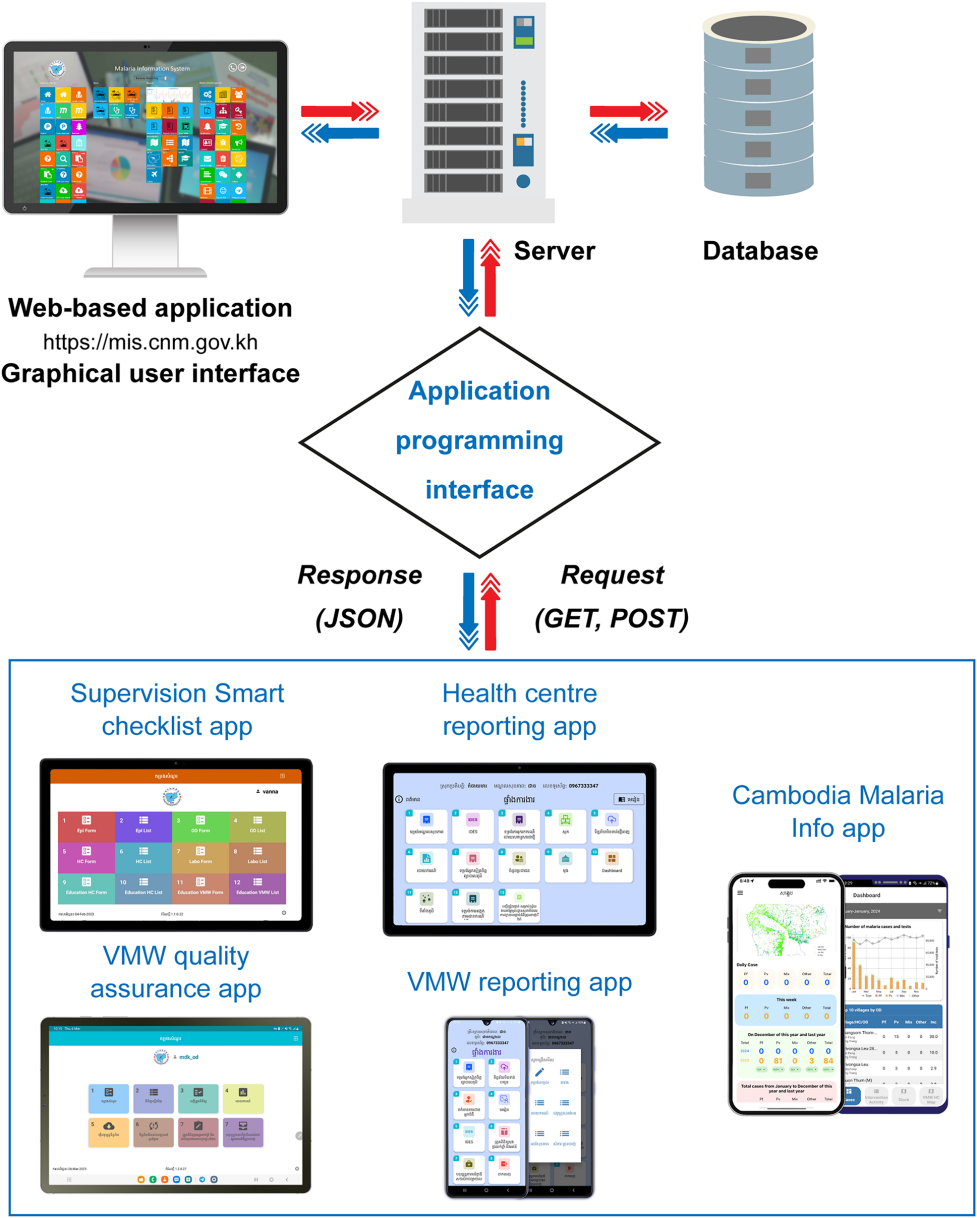
Malaria Information System (MIS) architecture. The MIS consists of a web-based application, two mobile apps for real-time malaria case management, one for health facility staff, and one for VMWs, two mobile apps to support supervision and quality assurance for VMWs and health centres and a publicly available app that provides daily updates on the malaria situation in Cambodia. Source: CNM/MIS

The system consists of a web-based application, two mobile apps for real-time malaria case management, one for health facility staff, and one for VMWs, two mobile apps to support supervision and quality assurance for VMWs and health centres, and a publicly available app that provides daily updates on the malaria situation in Cambodia (Fig. 2). The MIS system upgrade, including the mobile applications, was started in March 2017 and launched in October 2017. The application development architecture is fully open source, so the design and implementation of the software system, including its source code, are freely available for anyone to use, modify and redistribute (contact the corresponding author).

The web-based application uses a multi-tier architecture (Fig. 2). The server-side business logic is implemented using PHP, handling client requests, database interactions and server responses. This cross-platform, open-source programming language can be embedded within hypertext mark-up language (HTML) and improves website performance by reducing load times; a key consideration where internet connectivity may be limited. Data are stored in a Microsoft SQL Server, providing structured storage, report generation through stored procedures and query execution.

Native Android mobile applications were developed using Java in Android Studio. Google Maps application programming interface is used to create interactive maps at the village level. The cross-platform Firebase Cloud Messaging solution provides a mechanism for two-way communication through Cloud SMS messaging. Android applications are integrated with the web-based MIS through a JavaScript Object Notation (JSON) application programming interface, enabling two-way data transfer between the mobile client and the central server. Data entered via the mobile app are transmitted to the central MIS database, while data requests for maps, graphs and reports are processed on the server and returned to users in JSON format. Data transfer between client and server is handled over HTTP with dedicated endpoints configured for different data requests.

#### Graphical user interface

**Fig. 3:**
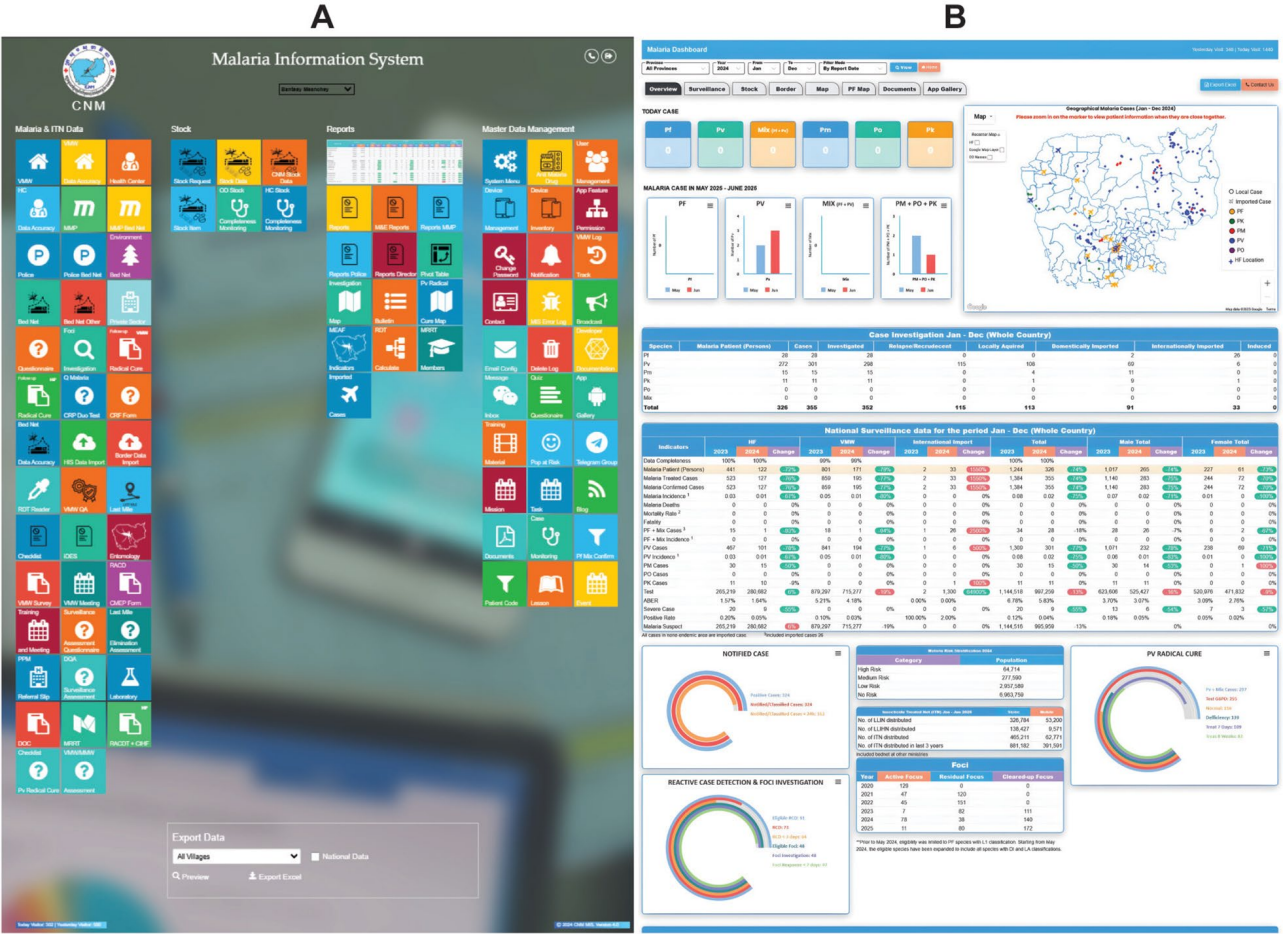
Malaria Information System (MIS) (**A**) graphical user interface, and (**B**) dashboard. The updated MIS prioritises a responsive, user-friendly interface to ensure accessibility and the dashboard provides automatically generated key analytics. The real-time publicly available version of the dashboard is available at: https://mis.cnm.gov.kh/index.php/Dashboard. Source: CNM/MIS

The updated MIS prioritises a responsive, user-friendly interface to ensure accessibility for users with varying levels of digital literacy (Fig. 3A). Developed using HTML and Bootstrap, the web-based platform features a clean layout with intuitive navigation across the login page, dashboard, reporting pages and customised interactive buttons. Functionality is organised into modules, displayed according to user level, allowing individuals to quickly access relevant tools without unnecessary clutter. The system uses the JavaScript charting library Highcharts to create interactive and responsive data visualizations including a wide range of chart types, including line, bar, column, area, pie, scatter, spline, heatmap, bubble, gauge and time-series charts. This is integrated with the Google Maps application programming interface (API) for detailed geospatial mapping down to the village level. Interactivity and data validation are enhanced using jQuery, preventing errors and improving the overall user experience. The mobile interfaces for VMWs and health centre staff follow the same design principles, with functionality tailored to their specific roles.

A key element of the MIS is its dashboard (Fig. 3B), which presents actionable programmatic information to stakeholders at all levels through accessible graphs, charts and maps. These visuals are based on data extracted from multiple tables within the central database. Because real-time retrieval and consolidation can be slow, a Windows-based utility (MIS Daily Task.exe), developed in C#, was introduced to enhance performance. This tool transforms database content into preformatted JSON files, which are cached nightly via Windows Task Scheduler, reducing reliance on live queries and significantly improving dashboard load times.

Additionally, MIS aggregated data at the operational district level are integrated with the national Health Management Information System (HMIS) with automatic data exchange via API. This eliminates replication of effort, with the MIS providing key malaria indicators to support strategic decision-making within Cambodia's Ministry of Health. The integration with HMIS has also increased the potential of the MIS platform to be used for other diseases, highlighting its value as a comprehensive and integrated disease surveillance and response system.

### Operational infrastructure and capacity building

MIS functionality and sustainability depend on several core operational components, including standardized training, mechanisms for managing and maintaining devices and tools for monitoring the availability and distribution of essential commodities. These elements provide the necessary infrastructure to support consistent system performance and reliable data collection across all health system levels.

#### Deployment and coverage

**Fig. 4:**
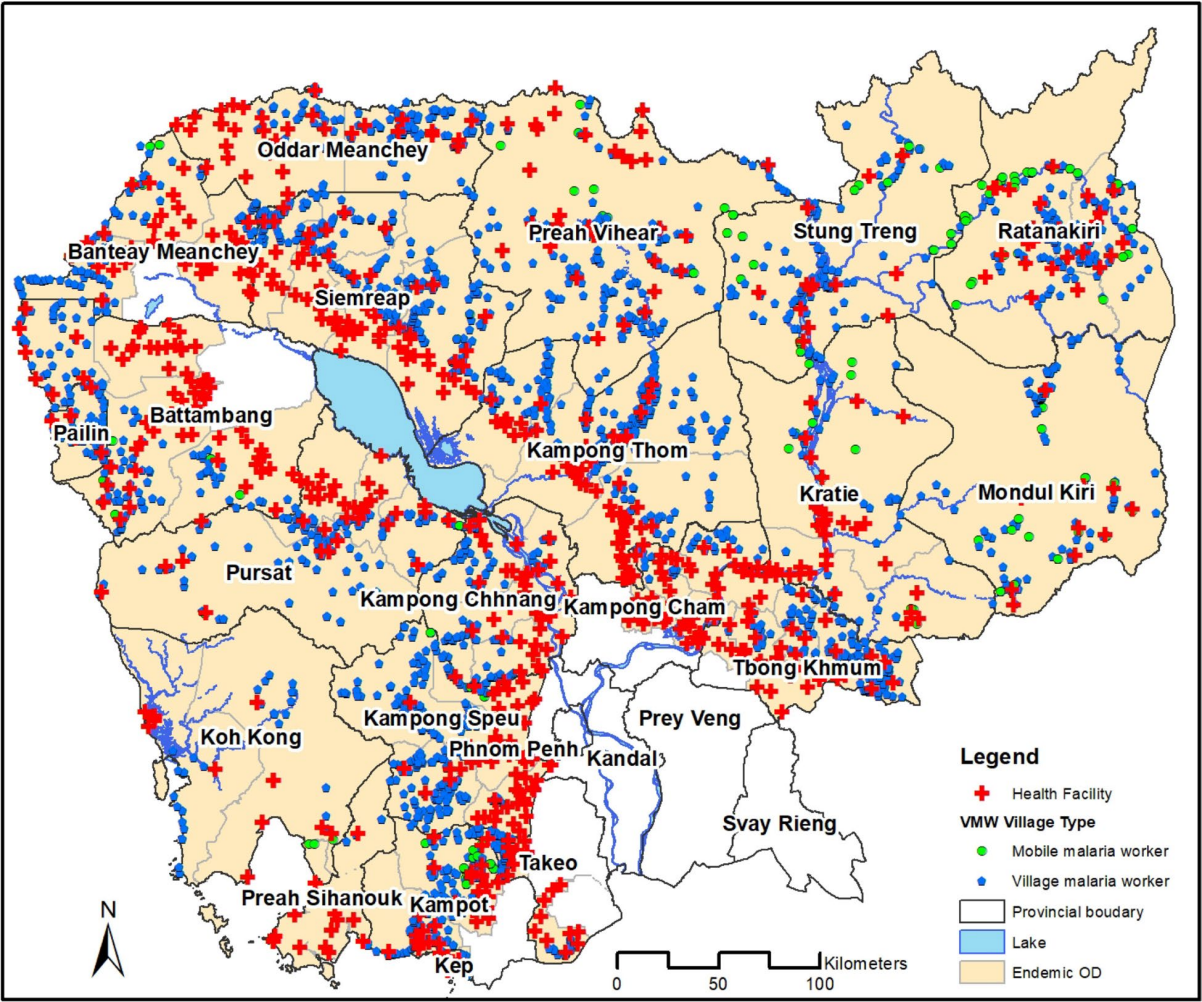
Coverage of the MIS surveillance network. By April 2018, 6200 VMWs from 3170 villages and 1632 health facility staff from 816 health facilities had been trained in the use of the MIS and mobile applications. Source: CNM/MIS

Following the rollout of the updated MIS in October 2017, and subsequent nationwide training efforts, the system was rapidly scaled to cover all malaria-endemic regions. By April 2018, 6200 VMWs from 3170 villages and 1632 health facility staff from 816 health facilities had been trained in the use of the MIS and mobile applications (Fig. 4). This widespread adoption enabled a rapid transition from a paper-based, retrospective, reporting system to a national real-time digital surveillance system to support malaria elimination. Initially smartphones and tablets were purchased and distributed via the programme with replacements issued in 2021 and 2023. However, to enhance sustainability, since 2023, the apps are installed on healthcare worker’s personal smartphones or general use devices at health facilities. This reflects the expanding access to these devices and improved connectivity in Cambodia.

#### Training and e-training

The clarity of the MIS user interface reduces the need for intensive training, with it taking approximately 3 h to train personnel at the national level, 2 h at the provincial level and 2 h at the district level. For the mobile applications, it takes approximately 4 h to train health centre personnel and 2 h for a VMW using a mix of face-to-face and e-training platforms. Initial training is delivered face-to face, with annual in-person refresher training delivered for the first two years following deployment to all personnel involved in surveillance, supported by e-training modules for reinforcement.

**Fig. 5:**
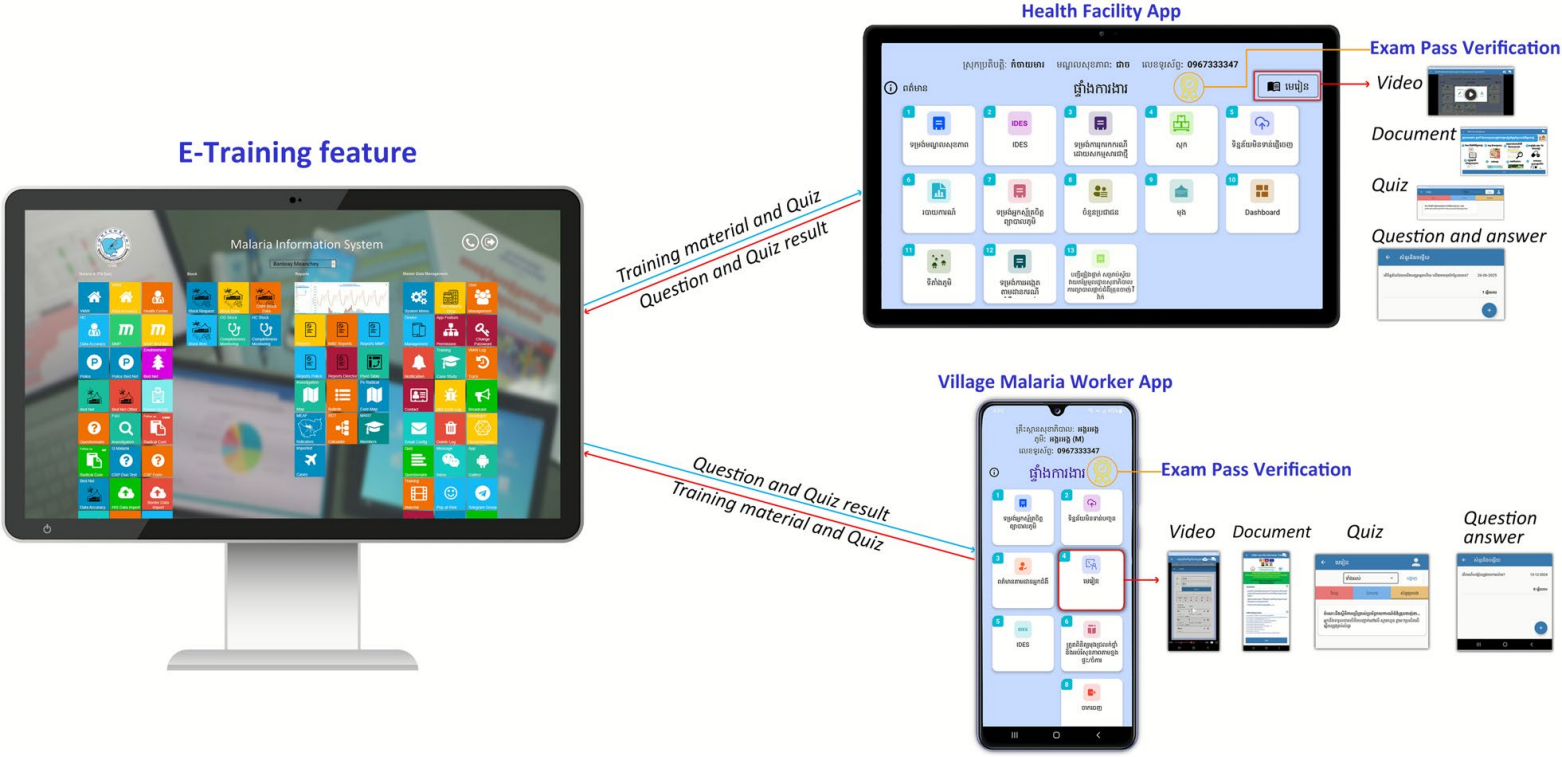
E-training module dataflow and content. Training materials are organised by category, including video tutorials, PDF guides and slide presentations. There is the facility to assign specific training modules to targeted users or user groups. Question and answer sections support user enquiries and clarify content while interactive quizzes assess use understanding and knowledge retention. On the dashboard, user training, progress and capacity status can be tracked and visualised. Source: CNM/MIS

For ongoing support and training, e-training platforms are incorporated into the MIS and mobile applications which can be accessed at any time. Training is modular and includes videos and presentations which can be completed at a pace that accommodates the learner, with the facility for questions and feedback, as well as quizzes for evaluating understanding (Fig. 5). Content is standardized across malaria diagnosis, treatment, prevention and the proper use of the mobile app for reporting. This digital approach enhances sustainability by allowing all personnel to be reached while eliminating travel expenses, reducing material requirements, reducing the administrative burden of arranging training and reducing the time taken away from work. It also facilitates access to healthcare workers in remote areas, ensuring they have up-to-date information. Training materials can be updated remotely and alerts and reminders issued to learners automatically. Progress is tracked automatically and can be assessed by supervisors, with notifications where training is incomplete or inadequate.

The e-training facility was especially valuable during the COVID-19 pandemic when travel restrictions and social distancing precluded in-person training. It allowed immediate updates and guidance on how to manage malaria cases in a rapidly evolving context while preserving the safety of both healthcare providers and patients. Additionally, skills could be preserved and normal services re-instated quickly once restrictions were lifted.

#### Data quality

Data accuracy is included as a specific module within the MIS. Data validation procedures are implemented at both the system and field levels. Client-side data validation uses JavaScript to prevent incorrect data entry, e.g., required field checks to drive completeness, format validation for dates and number range validation to flag unfeasible values, as well as drop down option lists to drive standardization. In addition, the central MIS team conduct monthly supervision field visits to health centres and VMWs flagged for anomalous data trends. During these visits, logbooks are reviewed and compared against MIS entries, with discrepancies documented and investigated to ensure data accuracy. To support this process, two mobile apps were developed, one to evaluate VMW performance and a supervision smart checklist app which can be completed digitally during field visits and uploaded to the MIS remotely. A geolocated standardized report is generated automatically, accessible within the MIS to any supervisor at any time, minimizing the administrative burden, ensuring consistency and enabling longitudinal performance and progress tracking.

#### Geolocation

**Fig. 6:**
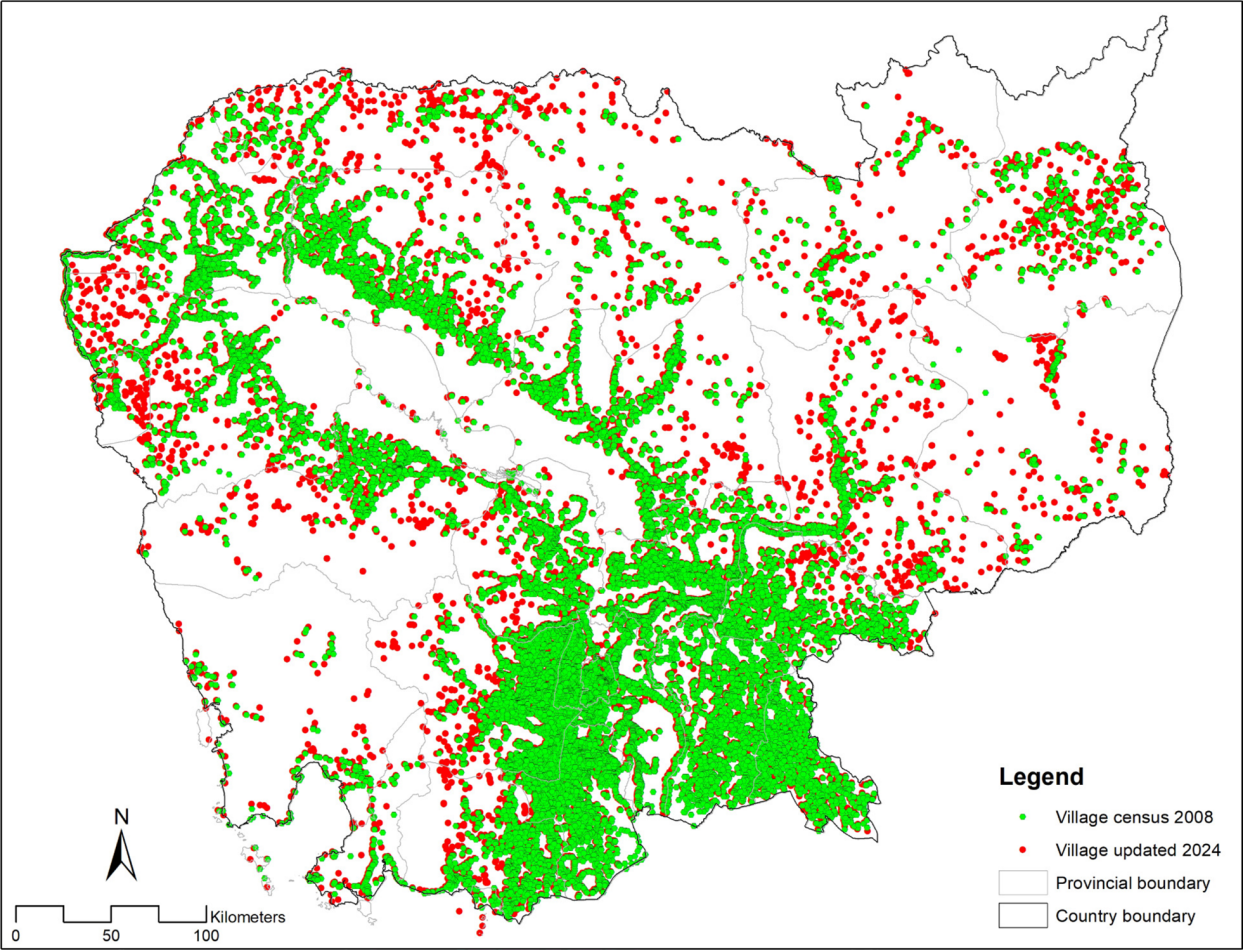
Geolocation of villages. The location of villages in the 2008 census versus geolocated villages within the MIS in 2024 verified by participatory mapping activities with health facilities. Source: CNM/MIS

An important advance has been the accurate geolocation and coding of villages, health facilities and operational districts – previously this information was derived from the national census data collected at 10-year intervals. Geolocation updates are included as quality control procedures within the MIS and annual participatory mapping activities are conducted at health facilities (Fig. 6). This resource-light process involves local health staff using their knowledge of the area to review village locations on Google Map, correct mistakes and add missing sites. Initially rolled out to malaria endemic regions, the initiative was so successful that it was extended to include malaria non-endemic regions to support national health interventions. Population information is maintained through data uploaded from the Commune database of the Ministry of Planning, with harmonisation of location codes between the two systems enabling data continuity and automation.

#### Device management

A critical MIS feature is the device management system. This controls access to the MIS from smartphones, tablets, laptops and desktops, allowing system administrators to monitor device usage, track device locations, manage connectivity and remotely control feature access or disable devices when necessary. It also allows application updates to be seamlessly distributed simultaneously throughout the user network. This functionality plays an essential role in maintaining data quality by ensuring all devices are using the correct software and operating as expected, as well as addressing any issues and guaranteeing that only approved personnel are entering data. It also allows rapid, automated hardware inventory checks to support financial management and reporting to donors and partners.

#### Stock management

**Fig. 7:**
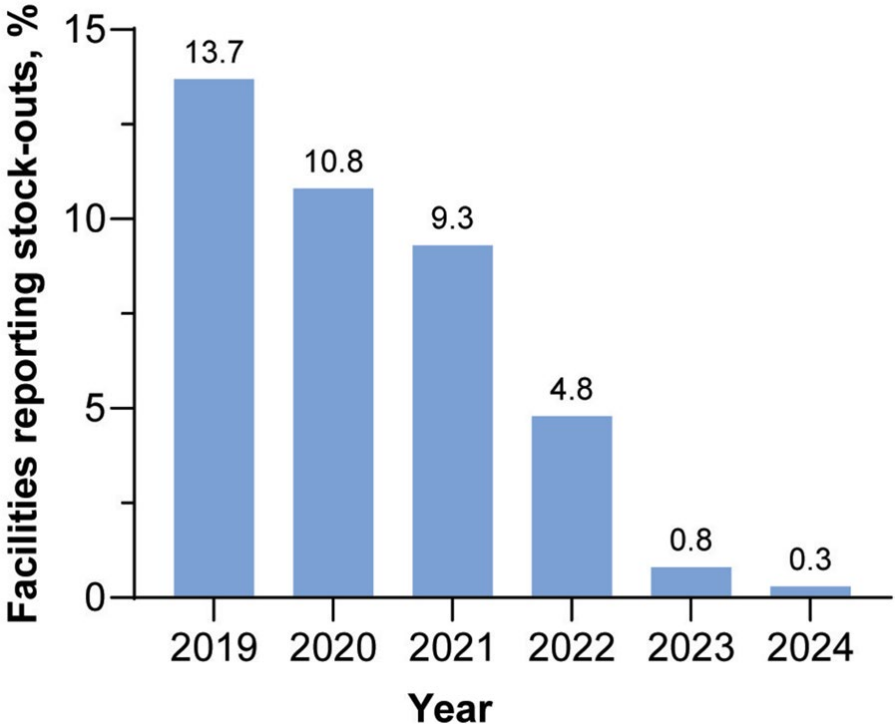
Proportion of facilities experiencing stockouts over time. The MIS incorporates a stock management feature for monitoring the availability of malaria-related commodities and proactively flags potential stockouts. Source: CNM/MIS

The MIS incorporates a stock management feature, enabling staff at the district and national levels to monitor the availability of malaria-related commodities, such as rapid diagnostic tests, treatments and insecticide-treated bed nets at the health facility level. This feature proactively flags potential stockouts, supporting a continuous supply of essential health products. The number of stockouts has reduced over time, being less than 0.3% of facilities in 2024 compared with 13.7% in 2019 (Fig. 7).

#### Vector control

Tracking bed net distribution and expiry dates is a key programmatic activity supporting malaria elimination. From the outset, a specific module was included in the MIS for this function, allowing geospatial visualisation of bed net distributions, highlighting where and when nets need to be distributed or replaced, aligned to malaria risk stratification and real-time incidence. To refine this, modules to manage bed nets for high-risk, mobile populations were added, including the police, military and forest workers. This saves time and resources by closing gaps in protection and ensuring that procurement and budgeting activities correspond to need.

### Surveillance for elimination

#### Real-time malaria case reporting and management

In Cambodia, most malaria cases are diagnosed and treated in the community by VMWs, with referrals to health centres for *P. vivax* relapse prevention. To support this workflow, two mobile applications, one for VMWs and one for health centres, facilitate malaria case reporting, patient data entry and follow-up of *P. vivax* patients receiving radical cure, including monitoring for adherence and adverse events. The health facility version includes additional modules for case investigation, reactive case detection, population demographics, VMW coordination, village catchment mapping, bed net distribution and real-time stock management. Both versions include dashboards for localised data visualisation and support automated, no-cost messaging for time-sensitive notifications. The VMW version is streamlined but retains key features for patient management and local data use.

**Fig. 8:**
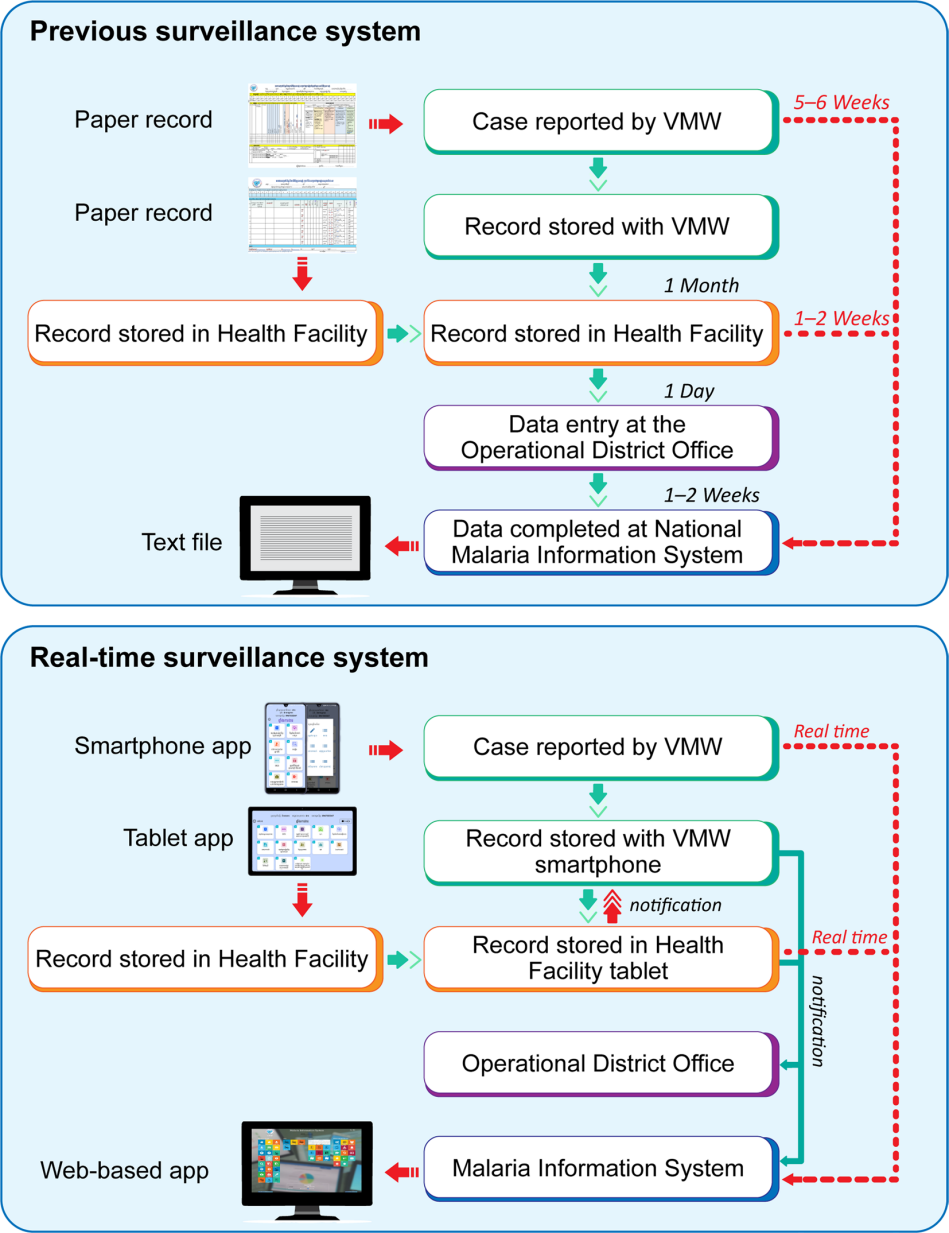
Reporting flow and timeliness. Real-time reporting with the updated MIS versus the previous paper-based system which could take 5–6 weeks for data to reach the national level. Source: CNM/MIS

**Fig. 9:**
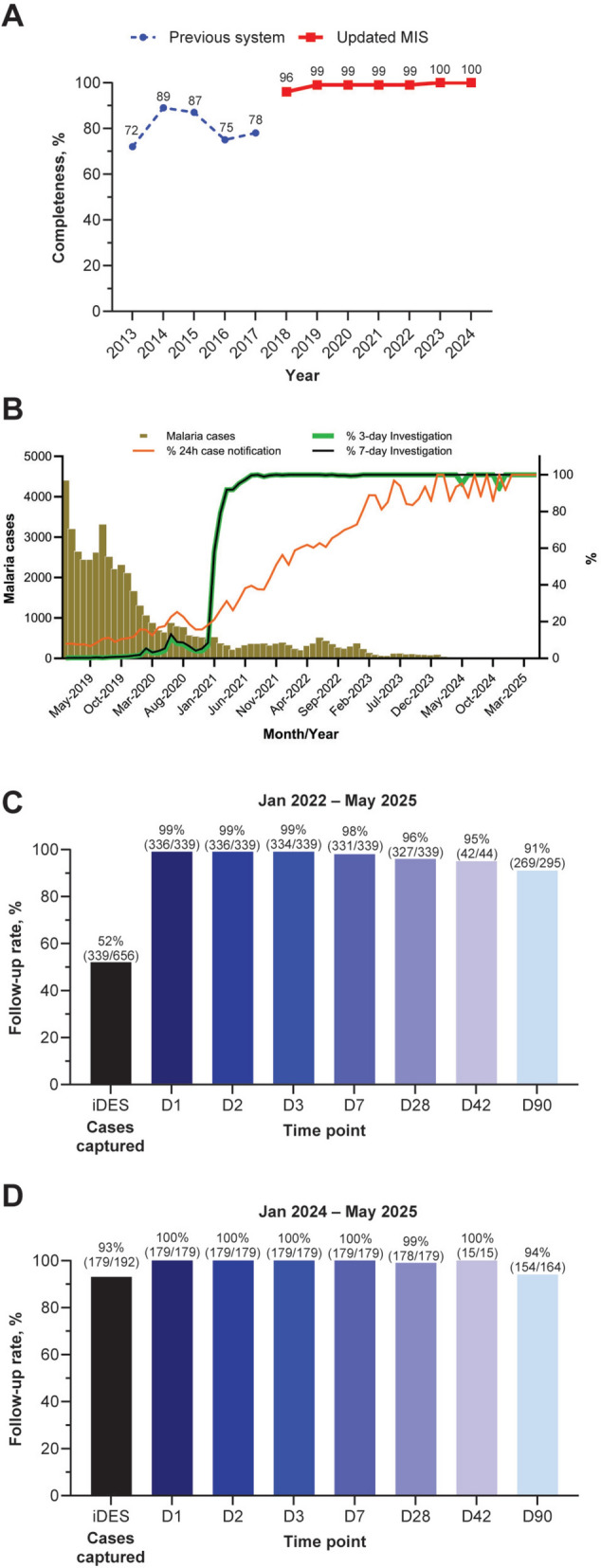
Key performance indicators. **A** Data completeness over time. **B** Proportion of 1–3–7 strategy targets achieved over time. Note that the variability in percent increases as the case incidence becomes very low. **C** Proportion of iDES targets achieved since inception in 2022. **D** Proportion of iDES targets achieved in 2024 − 2025. iDES follow-up is to day 42 (D42) for *P. falciparum* and to day 90 (D90) for *P. vivax*. Source: CNM/MIS

A major advance is the improved timeliness and completeness of case reporting. Previously, data transmission from the field to the national level could take 5–6 weeks due to manual reporting through district and provincial channels. The current system enables real-time data submission directly from the field via the mobile applications (Fig. 8). In areas with limited connectivity, data can be entered offline and automatically uploaded when a connection becomes available, ensuring full geographic coverage, even in remote areas. These tools have strengthened community engagement and significantly improved data quality. Field users can verify their entries, increasing accountability and ownership. Unlike the previous centralized system with limited local feedback, the new approach has brought data completeness close to 100% for both VMWs and health centres (Fig. 9A). The transition from manual monthly reporting to real-time digital entry has also reduced administrative burden and enabled continuous data availability. This is essential for effective implementation of the 1–3–7 approach, and the MIS supports high compliance levels by generating immediate notifications for new cases, prompting timely investigation and response actions. Thus, the updated MIS enhances malaria elimination efforts by strengthening the efficiency of the surveillance–response cycle (Fig. 9B).

#### Integrated drug efficacy surveillance

As malaria case incidence declines, conducting standard therapeutic efficacy studies as per WHO guidelines becomes unfeasible. Integrated drug efficacy surveillance (iDES) was formally launched in 2018 by CNM and rolled out nationally in a phased manner between 2019 and 2020. The aim is to provide routine, real-time monitoring of treatment outcomes for both *P. falciparum* and *P. vivax* to determine if adjustments to drug policy are required or to flag sub-optimal treatment. The iDES module includes standardized patient follow-ups up to day 42 for *P. falciparum* and day 90 for *P. vivax*. As every patient has a unique identifier, repeated malaria episodes can be linked to the initial malaria episode and classified as recrudescence or reinfection and as relapse for *P. vivax*. Data recording is fully integrated with case reporting within the mobile apps and enhancements include automated alerts, dashboard visualizations and targeted supervision based on iDES trends. Although completing follow-up can be challenging, iDES targets have improved over time (Fig. 9C and D).

#### Context specific functions

In 2021, Cambodia initiated intensified elimination acceleration strategies, termed ‘last mile activities’, to address residual transmission in hard-to-reach communities in remote areas [11]. Mobile malaria workers, who perform the same functions as VMWs, can travel to areas where access to malaria services is limited. Other interventions include community engagement, active case detection and the detection and treatment of asymptomatic cases. The MIS provides the high quality surveillance data and accurate stratification to implement these last mile activities on time and directed at the correct targets. Additionally, data analysis and monitoring activities for these specific interventions were incorporated into the MIS to track progress, identify areas for intervention and adjust strategies [11]. This is key because Cambodia is the first country in the GMS to implement last mile activities and the accumulated data will be informative for other programmes’ elimination acceleration strategies [11].

There is also the facility to upload data from the National Malaria Programmes for Lao PDR, Thailand and Viet Nam pertaining to the border regions with Cambodia. To highlight the importance of these data, specific analytics for border areas are incorporated into the MIS dashboard and accessible by neighbouring countries. Such analyses inform areas where collaborative efforts may be necessary to contain an outbreak, for example, or where cross-border community engagement needs to be strengthened to address persistent transmission.

#### Malaria reference laboratory

The Cambodia Malaria Reference Laboratory plays an essential role in malaria elimination by standardizing testing methods, providing training and overseeing quality assurance and quality control. These functions are integrated within the MIS, allowing for real-time tracking of resources and training. For example, CNM can continually assess the number of certified microscopists and their distribution across the country, and generate reports on supervision, slide cross-checking, training and assessments. The management of the national slide bank is also included, and key documents are available for reference, such as standard operating procedures and the quality assurance manual. Integration within the MIS supports diagnostic accuracy and facilitates data-driven decisions regarding resource allocation. Importantly, it also supports certification for malaria elimination which requires robust quality assurance systems to meet WHO requirements.

#### Entomological surveillance

In 2022, the CNM, with support from the U.S. President’s Malaria Initiative VectorLink project [21], developed an entomological surveillance module within the MIS. This module enables the collection and analysis of data on mosquito vector populations, insecticide resistance and the effectiveness of vector control interventions, such as indoor residual spraying and bed nets. Key aims were to support case-based entomological surveillance and strengthen foci investigations by identifying primary vectors to inform an appropriate response.

### Evidence-based policy impact

#### Actionable analytics for real-time policy response

The updated MIS provides real-time analytics designed to support both frontline operations and higher-level policy interventions. Accessibility is a core feature, with the dashboard offering automated, pre-specified outputs that allow users at all administrative levels to monitor trends, generate reports and visualize data without requiring technical expertise. Filters based on geography (from province down to village), time frame, species and other indicators enable tailored outputs, supporting fine-grained spatial analysis to the village level and local decision-making. Dashboard tabs for quick reference include an overview, surveillance, stock, border malaria, maps, a document library and an app gallery. The publicly available Cambodia Malaria Information mobile app further enhances transparency and engagement, particularly for community outreach.

For more advanced needs, a customizable analytics tool, functionally similar to Microsoft Excel pivot tables, is available to authorized users. This tool supports complex, user-defined queries and visualizations, enabling detailed exploration of case data, programme performance and intervention outcomes at both local and national levels.

The system's design supports real-time policy interventions at the national and district levels. National malaria programme staff can monitor malaria trends from the provincial down to the village level, using maps and graphs embedded within the MIS dashboard. This functionality enables rapid, data-driven decisions and timely intervention deployment. The district and provincial levels can quickly identify high-burden health centres or villages within their jurisdictions, which they can then prioritize during monthly coordination meetings with provincial health departments. An important function is that the MIS also allows monitoring and evaluation of interventions, again in real-time. This aspect ensures that successful interventions can be built on and deployed in other localities, while interventions that are working less well can be modified or re-evaluated.

#### Stratification

**Fig. 10:**
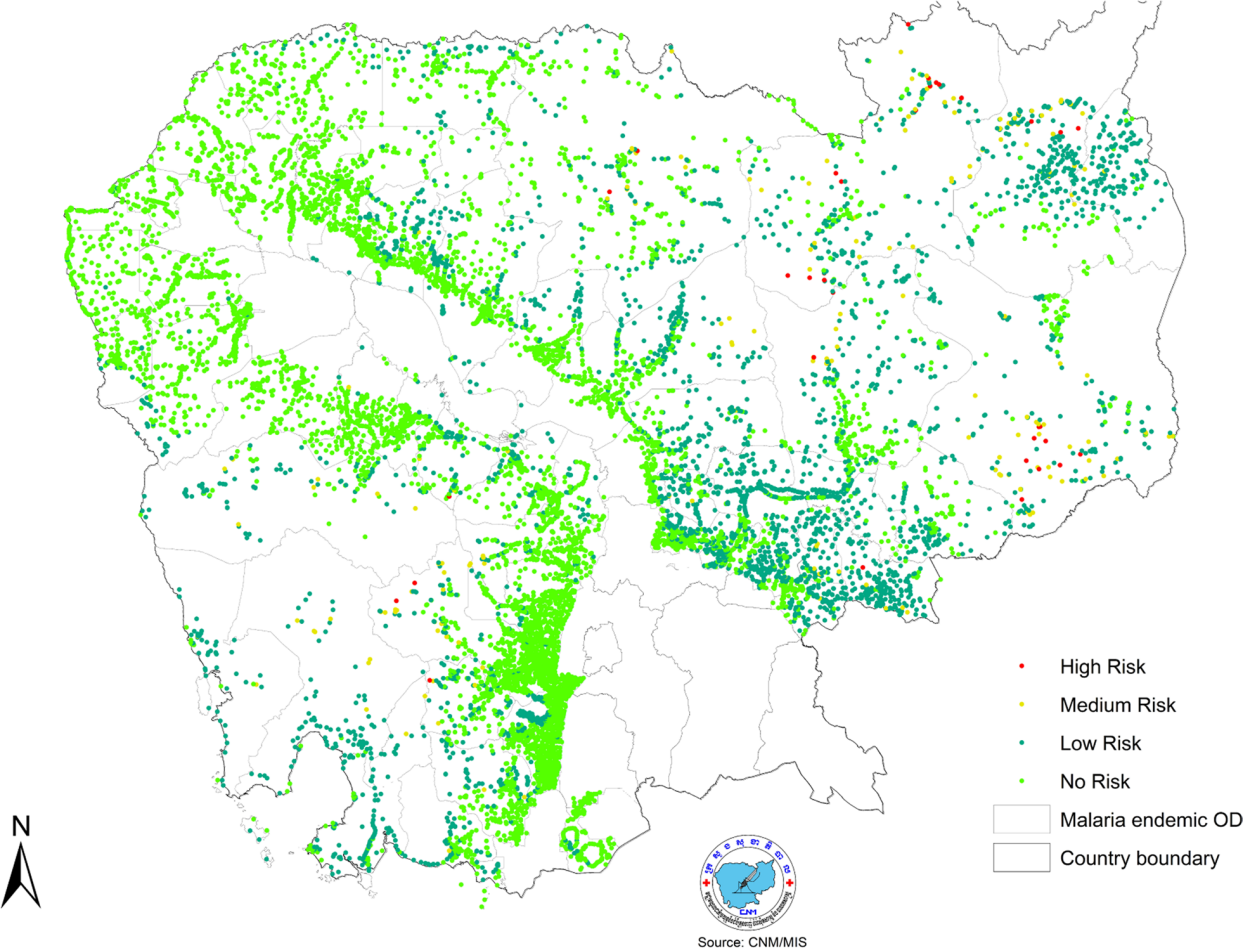
Real-time, incidence-based malaria risk stratification. The updated MIS enables incidence-based risk stratification by integrating geolocation, historical data, and real-time malaria incidence; risk maps are automatically and instantly updated to support dynamic resource reallocation based on current malaria risk levels. Source: CNM/MIS

Malaria risk stratification in Cambodia was previously based on an ecological premise considering proximity to forested areas and was a complex endeavour, taking several months, conducted annually. Although acceptable when case numbers were substantial, this method was not responsive to the heterogeneous and dynamic situation as malaria incidence declined. The updated MIS facilitated the transition to incidence-based risk stratification by combining geolocation, historical data and real-time malaria incidence. Risk maps can be updated automatically and instantly, allowing strategic resource reallocation depending on the current malaria risk level (Fig. 10). For example, stratification informs decisions on where ‘last mile’ targeted activities need to be implemented, commodities procurement and distribution and VMW placement. It also supports National Strategic Plan development and funding proposals to donors by demonstrating targeted and effective resource use. Stratification will continue to be a key activity to support the prevention of re-establishment of transmission.

#### Pilot implementation and operational research

Specific CNM research projects can be incorporated into the MIS. For example, as malaria is locally and eventually nationally eliminated, there is a risk that the VMW role in the community will be undermined. This is concerning, as this role is essential to maintain surveillance for imported/introduced cases and prevent re-establishment of transmission. A research programme integrated in the MIS evaluated expanded roles for VMWs delivering services for all febrile patients, using novel rapid diagnostic tests for dengue and bacterial infections [22]. This could retain the essential malaria surveillance function post-elimination and deliver better health and more rational antibiotic use [22]. Leveraging the MIS to deliver data collection, management and analysis for such studies enables efficient and rapid evaluation of novel approaches, supporting innovation.

#### External reporting and data sharing

The MIS supports external reporting needs by providing real-time indicator data to donor agencies, including the United Nations Office for Project Services and the Global Fund. The WHO World Malaria Report also publishes consolidated annual data from the MIS, eliminating redundancy in data gathering. Additionally, data from the MIS are integrated with the WHO Malaria Elimination Database for the GMS with automatic data exchange by API, contributing to regional quarterly reports, fostering collaboration. Data are also shared during joint initiatives with neighbouring countries to address cross-border malaria; an important issue across the GMS.

## Discussion

The Cambodian MIS represents a significant advance in disease surveillance, demonstrating how locally developed, digital platforms can enhance public health responses in resource-limited settings. The MIS has addressed long-standing challenges in timeliness, data completeness, usability and accessibility. The mobile applications function offline and synchronise automatically when connectivity returns, enabling frontline workers to submit real-time data, essential for responding to transmission foci, particularly in remote regions.

Beyond surveillance, the Cambodia MIS incorporates key operational features, including training and supervision, intervention monitoring, device management and stock management, supporting efficient resource use while minimizing administrative effort and overlap. These elements contribute to sustainability of the surveillance system and the overall malaria programme.

During the MIS development phase, one of the main challenges was building confidence in the feasibility of a locally developed solution. Understandably, there was an expectation from donors to consider established platforms such as DHIS2. There were also concerns around managing smartphones and tablets and whether training thousands of VMWs to report data through a mobile application was feasible. However, developing strong in-country capacity is key to maintaining operational relevance and sustainability. In-house development by CNM resulted in an elimination ready surveillance system with a user-friendly interface requiring minimal training for effective community-based reporting. The challenge of managing devices was overcome with a bespoke module which tracks, maintains and updates units remotely. Importantly, rapid iterative development was possible, for example, adding models for entomological surveillance, research studies or rolling out new processes and training to maintain malaria services during the COVID-19 pandemic. Thus, local development provided a deeper understanding of user needs and allowed flexibility in adapting the system to changing operational realities.

A key strength of the MIS is the promotion of decentralized data engagement. In the context of Cambodia, it was recognized early-on that a top-down approach could not deliver malaria elimination; the training and deployment of the extensive VMW network was a key turning point. However, fully leveraging this network required real-time data and accessible analytics. The integration of mobile apps allows direct interaction of VMWs and health centre staff with their own data through intuitive dashboards and user-friendly analytics tools, without needing formal analytical training. This capability enhances accountability, facilitates flexible local decision-making and fosters a culture of evidence-based practice across the health system.

The difference between disease surveillance and surveillance for elimination is key. For example, the WHO malaria module in DHIS2 relies on data aggregation at the health facility level often on a monthly or weekly basis, with case-based options as add-ons and analytics available to high-level decision makers; there is no response or follow-up functionality [23]. For example, in South Africa, only 61% of cases were notified within 24 h and data completeness was inadequate to fully support case classification [24]. In an elimination setting, where transmission is focal and case incidence low, case-based surveillance must be conducted in real-time and have high geospatial granularity [17, 25]. Moreover, surveillance will generate greater impact if field workers can access the data and tools needed for rapid response [17, 25]. For instance, in Cambodia, programme staff can monitor follow-up for *P. vivax* radical cure and coordinate resource allocation in response to outbreaks. These tools strengthen programmatic agility and responsiveness.

Stratification can be a time-consuming and complex task [26–28], but is essential for targeting interventions, identifying gaps in provision and finding remaining transmission foci. Integrated geospatial and time-series analytics support real-time, automatic risk stratification within the MIS, allowing rapid evaluation for adaptive planning at all levels. An important opportunity lies in integrating the MIS with the VillageView Platform to enhance planning for the prevention of malaria re-establishment. VillageView profiles are uniquely identified by a standardized code and official name, and compile essential data such as population size, forest coverage, proximity to rivers and water bodies and distances to major roads, markets and administrative centers. These profiles also include satellite-derived geospatial and imaging data, along with land use characteristics and precise location coordinates. By linking this standardized village-level data with the MIS, ongoing efforts to harmonize data registries can better support risk evaluations related to malaria importation and receptivity. This integrated approach will enable more accurate spatial analysis, strengthen surveillance and support evidence-based decision-making for targeted interventions and resource allocation.

At present, the MIS is the only health information system in Cambodia providing geolocated, case-based reporting in real-time at both community and national levels. While the system currently focuses on malaria, its architecture is designed to accommodate additional diseases with relative ease. However, despite these achievements, the system faces key limitations. The national MIS support team comprises just seven staff, constraining technical assistance and the capacity to manage a growing user base. To ease reporting burdens, artificial intelligence is being explored for automated rapid diagnostic test reading and facial recognition for patient identification. Expanding multilingual support would also improve accessibility for ethnolinguistically diverse communities. While the system performs well for retrospective and real-time analysis, it lacks predictive capabilities and integrating robust forecasting tools, potentially using machine learning, would enhance forward planning and early warning. Further developments should enhance interoperability with other national health systems, such as supply chain platforms, disease surveillance tools, and electronic medical records, enabling cohesive digital health system integration. For example, the Cambodian HMIS is currently transitioning to DHIS2, and to avoid the introduction of parallel systems, the MIS will need to be fully integrated with the new platform. Ongoing investment in the MIS is required to continue its development, adaptation and wider application. Strengthening coordination and ensuring that country-led systems are prioritized will be key to sustaining and expanding MIS impact to other sectors of the health system.

Several countries have adopted malaria case-based surveillance systems, though meeting all the requirements for elimination remains challenging [17]. In 2018, Lao PDR, with a context resembling Cambodia, launched an elimination-focused system featuring a dedicated web platform integrated into the national health system, with reporting shifting from paper to mobile phones [29, 30]. Thailand introduced an electronic malaria information system in 2011, with mHealth technology deployed in 2020 to enable direct data entry via mobile devices [31]. However, both Lao PDR and Thailand have not fully decentralized decision making, potentially due to the more limited role of VMWs. In Brazil, the SIVEP-Malaria system serves as the primary surveillance tool, but field reporting is still done on paper and submitted to Municipal Health Departments, delaying data entry and reducing programmatic value [32]. Cambodia’s experience suggests that mobile applications could help address these challenges. However, during such transitions, there needs to be specific planning to avoid parallel systems, and careful consideration of the technical and financial sustainability of the overall system. For example, Myanmar’s pioneering case-based system, incorporating mHealth, encountered significant technological and financial barriers, including dependence on internet access, system integrity issues, the need for continued outside support and redundancy caused by parallel systems [33]. This demonstrates the need for a technically skilled local workforce, strong country ownership and user-friendly platforms that are robust and stable enough to function effectively in field conditions. It is important for all partners to take the country context into account when considering digital approaches. Not every context is suited to the same approach, and locally driven development is a key tool in implementing and sustaining appropriate solutions.

## Conclusion

The Cambodian MIS is a leading example of digital health innovation, showing how user-centred, locally developed tools can enhance disease surveillance and public health response at all levels. With real-time data entry, decentralized analytics, and agile policy support, it has become central to Cambodia’s malaria elimination strategy. Sustained investment in local expertise has kept the system responsive, scalable and aligned with national goals. The system continues to evolve and future upgrades will focus on predictive analytics, interoperability and user support, broadening its role from elimination to preventing re-establishment and strengthening the wider health system through increased integration. Cambodia’s MIS demonstrates how context-driven digital transformation can drive sustainable, equitable health outcomes in line with national policies.

## Data Availability

All relevant data are available in the manuscript.

## Abbreviations

API: Application programming interface
CNM: National Center for Parasitology, Entomology and Malaria Control
GMS: Greater Mekong sub-region
HMIS: National health information system
HTML: Hypertext markup language
iDES: Integrated drug efficacy surveillance
JSON: JavaScript object notation
MIS: Malaria information system
PDR: People’s democratic republic
VMW: Village malaria worker
WHO: World Health Organization
